# GSAE: an autoencoder with embedded gene-set nodes for genomics functional characterization

**DOI:** 10.1186/s12918-018-0642-2

**Published:** 2018-12-21

**Authors:** Hung-I Harry Chen, Yu-Chiao Chiu, Tinghe Zhang, Songyao Zhang, Yufei Huang, Yidong Chen

**Affiliations:** 1 Department of Electrical and Computer Engineering, The University of Texas at San Antonio, San Antonio, TX 78249 USA; 2 Greehey Children’s Cancer Research Institute, The University of Texas Health Science Center at San Antonio, San Antonio, TX 78229 USA; 3 Department of Epidemiology & Biostatistics, The University of Texas Health Science Center at San Antonio, San Antonio, TX 78229 USA; 4 Laboratory of Information Fusion Technology of Ministry of Education, School of Automation, Northwestern Polytechnical University, Xi’an, 710072 Shaanxi China

**Keywords:** Gene superset analysis, Survival analysis, Deep learning, Autoencoder

## Abstract

**Background:**

Bioinformatics tools have been developed to interpret gene expression data at the gene set level, and these gene set based analyses improve the biologists’ capability to discover functional relevance of their experiment design. While elucidating gene set individually, inter-gene sets association is rarely taken into consideration. Deep learning, an emerging machine learning technique in computational biology, can be used to generate an unbiased combination of gene set, and to determine the biological relevance and analysis consistency of these combining gene sets by leveraging large genomic data sets.

**Results:**

In this study, we proposed a gene superset autoencoder (GSAE), a multi-layer autoencoder model with the incorporation of a priori defined gene sets that retain the crucial biological features in the latent layer. We introduced the concept of the gene superset, an unbiased combination of gene sets with weights trained by the autoencoder, where each node in the latent layer is a superset. Trained with genomic data from TCGA and evaluated with their accompanying clinical parameters, we showed gene supersets’ ability of discriminating tumor subtypes and their prognostic capability. We further demonstrated the biological relevance of the top component gene sets in the significant supersets.

**Conclusions:**

Using autoencoder model and gene superset at its latent layer, we demonstrated that gene supersets retain sufficient biological information with respect to tumor subtypes and clinical prognostic significance. Superset also provides high reproducibility on survival analysis and accurate prediction for cancer subtypes.

**Electronic supplementary material:**

The online version of this article (10.1186/s12918-018-0642-2) contains supplementary material, which is available to authorized users.

## Background

Nowadays gene set based analysis has been an essential step for interpreting gene expression data, for which a variety of bioinformatics tools have been developed to extract biological insights from different aspects. Among all methods, functional enrichment is the most common gene set based analysis to determine classes of genes that are associated with disease phenotypes, such as Gene Set Enrichment Analysis (GSEA) [1]. Function over-representation is another category for enrichment analysis, represented by The Database for Annotation, Visualization and Integrated Discovery (DAVID) [2, 3], among many others [4–6]. Researchers also employ gene set as a classifier; for example, the 50-gene PAM50 model was used to classify the subtypes of breast cancer [7]. Furthermore, many studies have conducted survival analysis at gene set level to predict clinical outcomes [8, 9]. Overall, gene set analysis improves the biologists’ capability to interpret functional impact to their experiment design. However, some studies have also disclosed the inconsistency of gene set results. Lau et al. showed that there are only minimal overlaps between the putative prognostic gene sets for non-small-cell lung cancer found in nine various studies [10]. Hence, inter gene sets association should be taken into consideration, as suggested by various studies, to limit inconsistency. While combined gene sets may provide consistency, its biological relevance is rarely discussed.

Deep learning methods have emerged recently in computational biology due to the increase of molecular and cellular profiling data. Convolutional neural network (CNN) methods were implemented for prediction of DNA-protein binding [11] or detection of phenotype-associated cell subsets [12]. Autoencoder, which is an unsupervised learning algorithm, was used for modeling gene expression through dimensionality reduction in many studies [13–15]. Lin et al. proposed a supervised neural network model for single-cell RNA-seq data that incorporate protein–protein interaction (PPI) and protein–DNA interaction (PDI) information [13]. However, the prior biological knowledge was only utilized to improve the performance of dimensionality reduction and cell type-specific identification, and the influence of combining PPI nodes was not examined.

In this study, we proposed Gene Superset AutoEncoder (GSAE), a multi-layer autoencoder model that incorporates a priori defined gene sets to preserve the crucial biological features from combining gene sets in the latent layer. We introduced the concept of the gene superset, an unbiased combination of gene sets, with weights trained by the autoencoder, where each node in the latent layer is termed a superset. The goal of this study is to determine the functional or clinical relevance of the learned gene supersets from our model, where the model evaluates gene expression data at the level of superset. To achieve our goal, we used large-scale RNA-seq data sets from The Cancer Genome Atlas (TCGA) to test GSAE and investigate the top ranked gene sets in the statistically significant supersets. We demonstrated that gene supersets preserve sufficient biological information with respect to tumor subtypes and clinical prognostic significance. Our study also compared different neural network classifiers and the superset classifier showed high accuracy in cancer subtype prediction. We concluded that superset produces more reproducible results than single gene sets, provides robustness in cancer subtype classification, and has the capability to learn potential gene sets association.

## Methods

### Data sets in this study

For Pan-cancer (PanCan) analysis, we collected TCGA RNA-seq data that was organized by TumorMap [16], which contains 9806 samples in 33 cancer types. In addition to entire TCGA data, we also selected breast invasive carcinoma (BRCA) data with 1099 samples for characterizing network nodes. For survival analysis, lung adenocarcinoma (LUAD) with 515 samples were chosen. Furthermore, we used four data sets with sufficient survival information, LUAD, BRCA, lower grade glioma (LGG, 523 samples), and skin cutaneous melanoma (SKCM, 469 samples) to compare the reproducibility of supersets and gene sets. The expression profiles of all tumor RNA-seq in this study are in the Transcripts Per Million (TPM) unit and then log-transformed (logTPM = *log*2 (TPM + 1)), which are re-analyzed uniformly for all samples [16].

### Gene superset autoencoder

**Fig. 1:**
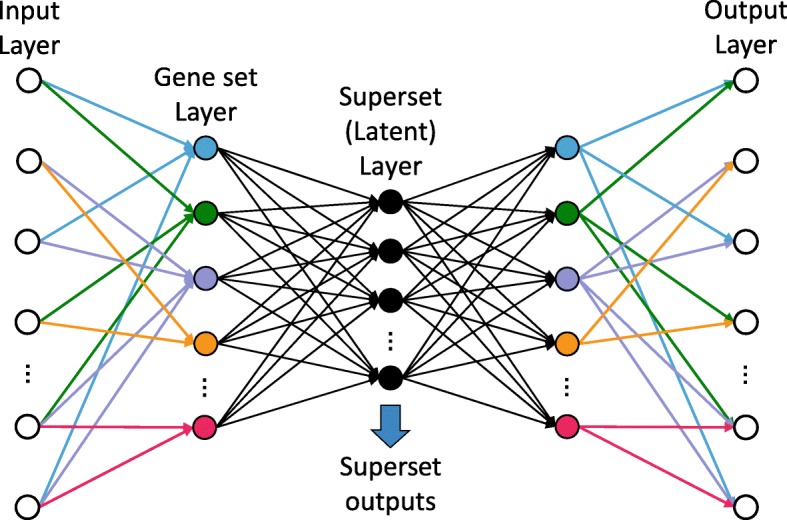
The architecture of gene superset autoencoder (GSAE). In the gene set layer, one color node represents a gene set, and edges in the same color show connect associate genes to a gene set

The architecture of GSAE is shown in Fig. 1. The input of the model is the gene expression profiles in log2 TPM values. The output *x* of the *j*th node in the *i*th layer can be formulated as

1 $$ {x}_{ij}=g\left({b}_{\left(i-1\right)}+\sum \limits_j{w}_{\left(i-1\right)j}{x}_{\left(i-1\right)j}\right) $$

where the bias *b* and the weight *w* are the two parameters that are learned in training, *g*() is the activation function, where we used the linear activation in the output layer and rectified linear unit (ReLU, defined in Eq. 2) in other layers to provide non-linearity while keeping a scoring feature in the model.

2 $$ ReLU=\left\{\begin{array}{c}x, if\ x>0\ \\ {}0, otherwise\ \end{array}\right. $$

Besides the input layer in our proposed autoencoder, the first two layers are the encoding network that extracts the important features from gene expression. ReLU activation mimics pathway activation/deactivation function, and comparing with with linear activation in all layers, GSAE with ReLU activation in the hidden layers has much better performance in cancer subtype prediction (data not shown). The decoder part comprises the last two layers; it is a complementary function of the encoder, and it aims to reconstruct the input from the converge layer. If the model is designed as a neural network-based classifier for cancer subtype prediction, the decoder network is replaced by a softmax function that is used as the output layer. At last, we choose the loss function to be either a mean square error function for the reconstruction loss, or a categorical cross-entropy function for multi-class classification error.

### Incorporate gene sets into the encoder layer

We designed the first layer in the encoder as the gene set layer, which incorporates the information of a collection of gene sets. Specifically, each node in this layer represents a gene set, where only genes in the input layer that belong to a gene set have connection to the node [13], and the weight for each connection is determined by the backpropagation in training steps. This is different from the fully connected layer commonly used in autoencoder. We adopted the chemical and genetic perturbations (CGP) collection downloaded from the Molecular Signatures Database (MSigDB) [1, 17] and replaced some highly dependent gene sets with a representative gene set among them.

From the output of the gene set layer, we can retrieve the gene set score of each input sample. Following with a dimension reduced dense layer, the superset layer (latent layer), can be used to investigate the combination of gene sets while keeping the crucial features needed to reconstruct the input data by the decoder. The superset layer is the key layer of our model, which we obtain a group of gene sets that correlate with divergence of cancer subtypes. Each node in this layer is considered as a superset, which is a combination of different gene set terms. In this study, we set the superset layer size to 200. With the information of supersets, we can analyze characteristics of the data set, such as the development of subpopulations or clinical relevance of a disease.

### Resolve dependencies among gene sets

The CGP collection in MSigDB includes the gene sets that represent expression signatures of genetic and chemical perturbations published in the literature. However, some gene sets are highly similar, and we need to mitigate the dependency. We used a similar method as in our previous study [18] to cluster gene sets with significant similarity. First, we omitted the gene sets that have less than 15 or more than 500 genes, which is also the default setting in original GSEA implementation [1]. We subsequently used kappa statistics to measure the similarity between all gene sets. We clustered gene sets with *P*-value < 10^−7^, and assigned the largest gene set as the representative of the cluster. At last, there were 2334 CGP gene sets including 18,107 genes selected to form the gene set layer.

### Establish and train the gene superset autoencoder

We implemented the model using Keras 1.2.2 (https://github.com/fchollet/keras) and used the custom layer method in Keras to accomplish the sparsity of gene set layer in order to keep the zero weights while optimizing the parameters. Since ReLU is used as the activation function, we selected He uniform initialization as the initializers for all layers [19]. To train the autoencoder, we used the stochastic gradient descent (SGD) optimizer that was revised in Lin et al. study [13], which was designed to deal with the optimization problem for sparse layers. The SGD parameters were set as following, *learning rate* = 0.05, *decay* = 10^− 6^, *momentum* = 0.9, and *Nesterov* = 1.

While training the model for a data set, we extracted 5% of data to be the validation set to avoid overfitting. With the *callbacks.EarlyStopping*() function in Keras, the model stops training when the loss of validation split does not improve in three consecutive epochs. At last, we imported the data set into the trained model and exported the outputs and weights of the encoder layers for further analyses in R.

### The use of additional machine learning tools

In this study, we have applied t-Distributed Stochastic Neighbor Embedding (t-SNE, https://cran.r-project.org/package=Rtsne) [20, 21], which has been widely used for dimensionality reduction. We performed t-SNE on superset results and embedded the high-dimensional data into a two-dimensional space, where potential subpopulations of the data were revealed. Another machine learning method, Hierarchical Density-Based Spatial Clustering of Applications with Noise (HDBSCAN, https://cran.r-project.org/package=dbscan) [22, 23], was used in the tumor subtype analysis. Comparing with many other clustering algorithms, HDBSCAN has good performance and stability in exploratory data analysis. We performed HDBSCAN on the t-SNE results to determine the possible clusters among the data. Ambiguous samples were classified as noise and omitted from further analysis.

### Evaluation of the clustering performance of t-SNE results

To compare the clustering performance of a t-SNE result, three index methods were used in this study: 1) Dunn index ($\frac{\min_{all\ \left(i,j,i\ne j\right)}{d}_B\left({C}_i,{C}_j\right)}{{\mathit{\max}}_k{d}_W\left({C}_k\right)}\Big)$, where numerator is the minimal between-cluster distance, and denominator is the largest within-cluster distance) (clv v0.3–2.1 in R); 2) Silouette index (the mean of the mean silhouettes through all the clusters) (clValid 0.6–6 in R); and 3) inter-intra distance (IID) index. Slightly different from Dunn Index, the IID index takes the ratio of mean over between-cluster distances to the mean over within-cluster distances. We also define *d*_*B*_(*C*_*i*_, *C*_*j*_) as the center-to-center distance, where cluster center is defined as the median of all samples within a cluster, and *d*_*W*_(*C*_*k*_) is defined as the distance of all samples within cluster *C*_*k*_ to the center of *C*_*k*_, or

3 $$ IID\  Index=\frac{1/{n}_B{\sum}_{all\ i,j;i\ne j}{d}_B\left({C}_i,{C}_j\right)}{1/{n}_W{\sum}_k{d}_W\left({C}_k\right)} $$

where *n*_*B*_ and *n*_*W*_ are the number of between-cluster pairs and the number of clusters, respectively.

### Differential superset analysis between tumor subtypes

After performing t-SNE on the superset layer outputs, we subsequently determined the subtypes of a data set by using HDBSCAN. To find the supersets with a subtype pattern, we compared superset values between one tumor subtype (group 1) and the other subtypes (group 2) by one-tailed Mann-Whitney-Wilcoxon *U* test (MWW) with a location shift of “*mu*” (*mu* was assigned to change the stringency of the test). Significant supersets (MWW *P*-value < 0.01) that have larger values in group 1 were named as up-supersets, whereas down-supersets were the significant supersets with larger than in group 2. We further investigated gene sets in the significant supersets. To quantify the contribution of *i*^th^ gene set in *j*^th^ superset, *gsScore* was calculated as following,

4 $$ {gsScore}_{ij}=\left({\mu}_1^{(i)}-{\mu}_2^{(i)}\right)\times {w}_{ij} $$

where *μ*_*1*_ and *μ*_*2*_ are the average of the *i*^th^ gene set values in the two groups, and *w*_*ij*_ is the weight in the model corresponding to the connection from the *i*^th^ gene set to the *j*^th^ superset. In up-supersets, gene sets with *gsScore* greater than a positive cutoff (in the right tail) were selected. In the opposite, gene sets in the down-supersets with *gsScore* less than a negative cutoff (in the left tail) were selected. Those gene sets are the potential high impact gene sets of the subtype (group 1).

### Kaplan-Meier survival analysis on superset layer

We examined whether GSAE retains survival related features. For each superset and gene set, we used a median split (median of the superset or gene set value) to create two groups and performed log-rank test. For each prognostic significant superset, we ranked gene sets according to the *gsScore* (Eq. 4) and further investigated the survival relevance of top gene sets.

## Results

### Cancer type information preserved in low dimension outcome

**Fig. 2:**
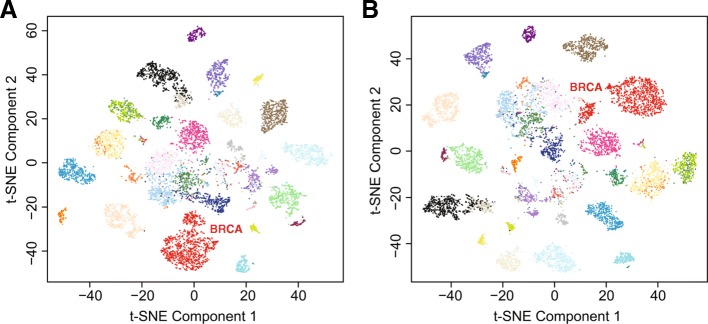
The t-SNE results of TCGA 9806 samples using (**a**) logTPM data with 15,975 genes (an initial PCA step was performed), and (**b**) 200 superset outputs

To test the capability of GSAE to retain crucial features in the superset layer, we used TCGA PanCan RNA-seq logTPM data, 15,975 genes selected with *μ* > 1 and *σ* > 0.5 across 9806 samples in 33 cancer types, as GSAE inputs and exported the superset layer results. We performed t-SNE on TCGA logTPM data and the superset layer outputs (200 nodes), and the results are shown in Fig. 2, in which the color of each node was labelled according to the cancer type information. The groupings of cancer types in the two t-SNE plots are nearly identical, where most cancer types form an individual cluster. The mingling of few cancer types are also similar in both figures. We used three index methods, Dunn index, Silouette index, and IID index, to evaluate the resemblance of the two t-SNE results in Fig. 2. Overall, with the input dimension reducing by more than 98%, it leads to 23.48% loss in the clustering performance between the two t-SNE results with Dunn index (Table 1). However, we got comparable clustering performance while using the other two index methods (− 0.85% in Silouette index and − 2.54% in IID index, respectively, Table 1). We concluded that the model is able to retain cancer type-associated features of a data while reducing dimensionality.

**Table 1:** Evaluation of the clustering performance of the two t-SNE results in Fig. 2. As a reference, the compression rate from 15,975 features down to 200 supersets is about 98.7%

Index Method	t-SNE of 15,975 genes	t-SNE of 200 supersets	Compression loss^a^
Dunn index	0.247	0.189	23.48%
Silouette index	0.355	0.358	−0.85%
IID index	7.924	8.125	−2.54%

^a^The compression loss = (index score of genes – index score of supersets) / index score of genes

### Indication of gene sets associated with breast cancer subtypes

**Fig. 3:**
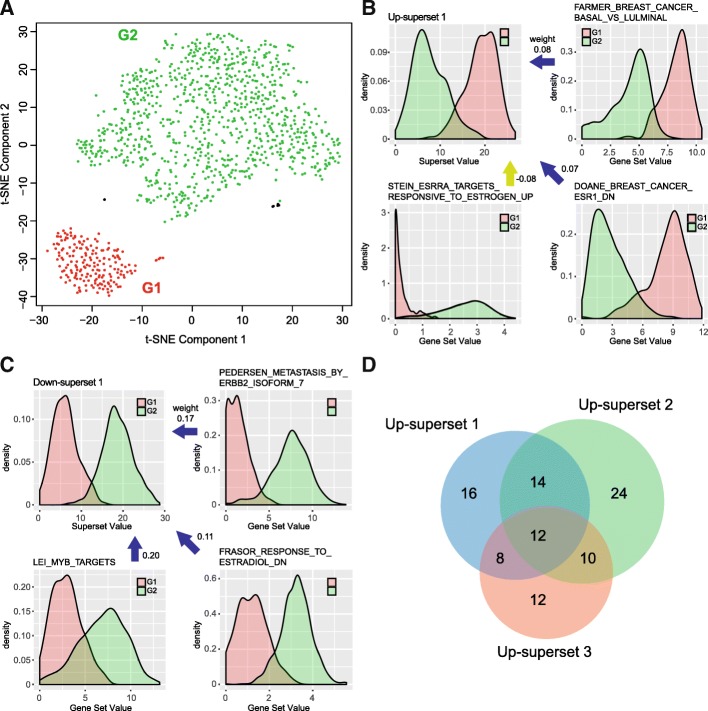
Subtype analysis in BRCA data set. (**a**) The t-SNE results of BRCA data, where HDBSCAN classified the samples into two groups. The noisy samples were labeled in black and omitted from further analysis. (**b**) The density plots of the most significant up-superset and three selected top gene sets. The blue/yellow arrow corresponds to positive/negative weight in the model between the gene set and superset. (**c**) The density plots of the most significant down-superset and three selected top gene sets. (**d**) The Venn diagram of the significant gene sets in the top 3 up-supersets

In Fig. 2, we learned that the samples labeled in red are separated into two clusters, and we further verified that they belonged to BRCA. We used GSAE to analyze the BRCA data separately to discover gene sets that are support this subtype differentiation. There were 15,183 genes in 1099 samples that meet the criterion of *μ* > 1 and *σ* > 0.5, where they were used as the model input. After training of the model, we exported the superset results and performed t-SNE, which is shown in Fig. 3a. We applied HDBSCAN, which clustered the samples into two groups, where group 1 (G1) is labeled in red and group 2 (G2) in green. The noisy samples defined by the algorithm were omitted. Four up-supersets and three down-supersets were determined (*P*-value < 0.01) using one-tailed Mann Whitney *U* test with location shift *mu* = 9, where only supersets with a huge difference between the two groups could pass the test. In each significant superset, those with *gsScore* > 2 *sd* (standard deviation of all *gsScores* in the superset) are the high impact gene sets of the superset. All high impacts gene sets of 7 significant supersets are listed in Additional File 1: Table S1, and the *PScore* (−log_10_(*P*-value)) of Mann Whitney *U* test (location shift set as 0.5) of each gene set was also included.

Top 15 gene sets in the most significant up-superset and down-superset are listed in Tables 2 and 3. The two superset density plots of gene set values (output of gene set nodes) in Fig. 3b and c show the vast difference between the two groups in those significant supersets. We also identified many high impact gene sets associated with breast cancer subtypes. For example, “FARMER_BREAST_CANCER_BASAL_VS_LULMINAL” clearly indicates the two groups are possible Basal and Luminal subtypes [24]. The study of “STEIN_ESRRA_TARGETS_RESPONSIVE_TO_ESTROGEN_UP” gene set also suggested that *ESRRα* might be a therapeutic target for triple negative breast cancer [25]. Group 1 has a higher value in “DOANE_BREAST_CANCER_ESR1_DN”, which matches the gene set condition where these genes were down-regulated in *ESR1* positive samples [26]. Genes that are involved in “PEDERSEN_METASTASIS_BY_ERBB2_ISOFORM_7” differentiates the HER2 positive and negative BRCA subtype [27]. A study has shown that *c-Myb* differed significantly across the subtypes, where Basal-like has the lowest expression [28], which fits the result of “LEI_MYB_TARGETS”. There is no direct connection of Estradiol with breast cancer subtype, but it is an estrogen and its target gene set “FRASOR_RESPONSE_TO_ESTRADIOL_DN” might be a potential subtype marker.

**Table 2:** Top 15 gene sets in up-superset #1 in BRCA subtype analysis

Gene Set Terms	*PScore* ^a^	*gsScore*	Weight^b^
CUI_TCF21_TARGETS_2_UP	81.999	0.980	0.198
PEDERSEN_METASTASIS_BY_ERBB2_ISOFORM_7	92.578	0.927	−0.154
GRADE_COLON_AND_RECTAL_CANCER_UP	68.314	0.427	0.138
DOANE_BREAST_CANCER_ESR1_DN	87.537	0.374	0.066
VANTVEER_BREAST_CANCER_ESR1_DN	80.186	0.366	0.083
HATADA_METHYLATED_IN_LUNG_CANCER_UP	76.111	0.333	−0.103
FARMER_BREAST_CANCER_BASAL_VS_LULMINAL	90.606	0.321	0.079
RICKMAN_TUMOR_DIFFERENTIATED_WELL_VS_MODERATELY_UP	80.636	0.247	−0.113
BOQUEST_STEM_CELL_DN	10.658	0.245	−0.130
BONOME_OVARIAN_CANCER_SURVIVAL_OPTIMAL_DEBULKING	54.414	0.228	−0.100
MOREAUX_MULTIPLE_MYELOMA_BY_TACI_UP	88.073	0.206	−0.059
YANG_BREAST_CANCER_ESR1_DN	71.886	0.194	0.088
DOUGLAS_BMI1_TARGETS_DN	18.273	0.177	−0.127
STEIN_ESRRA_TARGETS_RESPONSIVE_TO_ESTROGEN_UP	84.141	0.174	−0.076
BERNARD_PPAPDC1B_TARGETS_DN	88.410	0.161	−0.073

^a^The *PScore* of gene set Mann Whitney *U* test with location shift = 0.5

^b^The weight in the model corresponding to the connection of a gene set to the corresponding superset

**Table 3:** Top 15 gene sets in down-superset #1 in BRCA subtype analysis

Gene Set Terms	*PScore* ^a^	*gsScore*	Weight^b^
PEDERSEN_METASTASIS_BY_ERBB2_ISOFORM_7	92.578	0.997	0.166
VANTVEER_BREAST_CANCER_ESR1_DN	80.186	0.811	−0.185
LEI_MYB_TARGETS	55.903	0.800	0.201
DOANE_BREAST_CANCER_ESR1_DN	87.537	0.644	−0.114
CUI_TCF21_TARGETS_2_UP	81.999	0.511	−0.103
ACEVEDO_NORMAL_TISSUE_ADJACENT_TO_LIVER_TUMOR_DN	46.415	0.340	0.127
DELACROIX_RARG_BOUND_MEF	55.442	0.336	0.141
SENGUPTA_NASOPHARYNGEAL_CARCINOMA_UP	37.512	0.230	−0.074
FRASOR_RESPONSE_TO_ESTRADIOL_DN	77.02	0.218	0.108
HATADA_METHYLATED_IN_LUNG_CANCER_UP	76.111	0.215	0.066
SMID_BREAST_CANCER_LUMINAL_A_UP	51.385	0.211	0.077
FOSTER_KDM1A_TARGETS_UP	62.021	0.188	0.091
SHEDDEN_LUNG_CANCER_GOOD_SURVIVAL_A12	6.764	0.178	−0.096
SWEET_LUNG_CANCER_KRAS_UP	6.548	0.178	0.138
KOYAMA_SEMA3B_TARGETS_DN	24.534	0.176	0.094

^a^The *PScore* of gene set Mann Whitney *U* test

^b^The weight in the model corresponding to the connection of a gene set to the superset

After reviewing these gene sets, because the Basal subtype accounts for 15% of the breast cancer population, we hypothesized that G1, the small subpopulation in red in Fig. 3a, is the Basal subtype of breast cancer. We checked the TCGA clinical information and PAM50 classification results and verified that 156 of 175samples (with complete estrogen status or PAM50 subtype information) in G1 are either Basal-like or triple negative samples. This result demonstrates that our proposed superset autoencoder is able to reveal the subpopulation features and biological relevance.

We compared with GSEA results between G1 and G2, and 53 out of 124 (42.7%) high impact gene sets are also GSEA enriched gene sets (nom *P*-value < 0.05), which indicates the reliability of our results. To examine whether a superset contains some exclusive gene sets, we compared the top 3 up-supersets (Additional File 1: Table S1A-C) and the Venn diagram is shown in Fig. 3d. Many of the overlapped gene sets are associated with the Basal subtype (12 common gene sets in Additional File 1: Table S1, bold font). Up-superset 1 has additional estrogen related gene sets (Additional File 1: Table S1A, colored in blue); up-superset 2 holds some gene sets that are relevant to *ERBB2* (Additional File 1: Table S1B, colored in blue).

### Prediction of breast cancer PAM50 subtypes with superset classifier

To test if our model can be used as a classifier to predict cancer subtypes, we reconstructed our model to the architecture shown in Additional File 2: Fig. S1A, where the decoder network is replaced by a softmax function output (input – encoder – prediction output). With the clinical annotation organized by the UCSC Cancer Genomics Browser [29, 30] (captured in 2015, https://genome-cancer.ucsc.edu), we selected 821 BRCA samples with 15,183 genes in Basal, LumA, LumB, and Her2 PAM50 subtypes as input data to test the performance of the superset classifier (normal-like subtype was removed due to small sample size). Using 10-fold cross-validation to test the superset classifier, we achieved a good performance of 88.79% prediction accuracy.

With the same input, we also compared with four different neural network models, 1) gene set classifier, in which the superset layer is removed (Additional File 2: Fig. S1B), 2) 2-layer fully connected encoder network with the same size of the superset classifier (Additional File 2: Fig. S1C), 3) 2-layer fully connected encoder network, where the size of each layer was optimized by Hyperas [31] (Additional File 2: Fig. S1C), and 4) 4-layer fully connected encoder network, where the size of each layer was optimized by Hyperas (Additional File 2: Fig. S1D). The size and 10-fold cross-validation accuracy of each classifier are listed in Table 4. We have tuned the SGD parameter setting of each model in order to get the best performance.

**Table 4:** The size of encoder layers and the 10-fold cross-validation accuracy of each neural network classifier

NN classifier^a^	Input Type	Encoder Layer 1^d^	Encoder Layer 2	Encoder Layer 3	Encoder Layer 4	Accuracy of 10-fold cross validation
Superset	Genes^b^	2334	200			88.79%
Gene set	Genes	2334				87.69%
2-layer fc	Genes	2334	200			47.86%
2-layer fc	Genes	2000	500			37.98%
4-layer fc	Genes	2000	200	100	50	46.06%
2-layer fc	PC^c^	400	100			87.57%
4-layer fc	PC	200	200	100	25	87.57%

^a^2-, 4-layer fc: 2- or 4- layer fully connected AE

^b^Genes input is the 15,183 genes of TCGA BRCA RNA-seq data

^c^PC input is the top 500 principal components of PCA analysis

^d^The encoder layer 1 of superset and gene set classifier is the gene set layer (not a fully connected layer)

The prediction accuracy of gene set classifier (87.69%) is close to that of the superset classifier, which implies the gene set layer contains sufficient information for classification. On the other hand, all three classifiers with fully connected encoder have low prediction accuracy (< 50%, Additional File 2: Fig. S1C and D), mainly due to the large number of weights need to be trained to attain (or fail to attain) an optimal model. To alleviate the training burden, we reduced the input number by performing principal component analysis (PCA) on BRCA data first and selected top 500 principal components (PCs) to test the models with fully connected encoder (Additional File 2: Fig. S1C and D, layer size was also optimized by Hyperas, Table 4). The prediction results (87.57%) are equivalent to the superset classifier, indicating that the gene set layer and top PCs both preserve important subtype features. While both PC classifier and gene set classifier achieved the same accuracy, we can design our network to emphasize certain features (e.g. PAM50 subtype classification), based on the fact that we understand the biological functions of a priori defined gene set, and the flexibility of choosing different functional sets (signaling pathways, immunological signatures, etc).

We further tested the mean sensitivities and specificities of superset classifier by ten (10) iterations of 10-fold cross-validations (Table 5). We have near perfect specificity in all four BRCA subtype, especially in Basal (1.000) and HER2 (0.977). In addition, these two subtypes both have high sensitivity (Basal: 0.957 and HER2: 0.924). Prediction errors mostly occurred between Luminal A and Luminal B subtypes with relatively low sensitivities (0.862 and 0.835, respectively) while maintaining consistent specificities (0.935 and 0.907, respectively). This is expected due to the ambiguous cutoffs to define Luminal A & B at gene expression levels [32]. Overall, the superset classifier provides high sensitivity and specificity in BRCA subtype prediction.

**Table 5:** The mean sensitivities and specificities of superset classifier by ten iterations of 10-fold cross-validations

BRCA subtype	Sensitivity	Specificity
Basal	0.957	1.000
HER2	0.924	0.977
Luminal A	0.862	0.935
Luminal B	0.835	0.907

### Prognostic significance for lung adenocarcinoma

TCGA LUAD data set was employed to test if the model is capable of retaining survival related features in the superset layer. With the same gene selection criterion, 15,188 genes in 515 samples were used as the model input. We also organized the TCGA LUAD survival information to a 5-year survival record, where the maximum survival time was set as 1825 days, and a death event that occurred after five years was censored at 5 years. After performing log-rank test on the superset results, we determined 6 supersets with log-rank *P*-value < 0.001, which were considered as prognostic significant nodes. We ranked the gene sets in those six supersets by the *gsScore*, and the top 20 gene sets in each superset are listed in Additional File 3: Table S2. The top ranked gene sets that also showed significance in gene set log-rank test were selected to probe the biological relevance of lung adenocarcinoma.

**Fig. 4:**
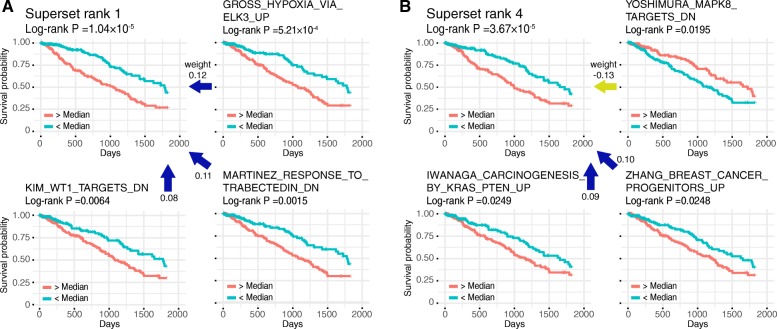
The Kaplan-Meier Curves of (**a**) 1st ranked superset and selected three top 20 gene sets associated with the superset, (**b**) 4th ranked superset and selected three top 20 gene sets associated with the superset. The blue/yellow arrow corresponds to positive/negative weight in the model between the gene set and superset

We picked the first and fourth ranked supersets as two examples, and the top 15 gene sets in the two supersets are listed in Tables 6 and 7. We chose the 4th ranked superset due to the least overlap of significant gene sets with the 1st ranked superset. We selected three gene sets tested significant by the log-rank test from the two supersets and plotted the Kaplan-Meier survival curves in Fig. 4. In rank 1 superset, several significant gene sets are related to the survival of LUAD. A study has shown that decreased mRNA expression of *TCF21*, a tumor suppressor, is a core predictor for poor prognosis in patients with lung cancers in two studies [33–35], agree with what we found the prognosis association from TCGA LUAD with gene set “CUI_TCF21_TARGETS_2_UP” (*P* = 1.30 × 10^− 4^). “KIM_WT1_TARGETS_DN” (*P* = 0.0064) is related to the oncogene *WT1* in lung cancer, and the high expression of *WT1* links to an unfavorable impact on the prognosis [36]. We also found some gene sets that no previous study showed direct connection with the prognosis of LUAD. Previous studies have revealed that ETS-related transcription factors are associated with non-small-cell lung cancers (NSCLC) [37, 38]. *ELK3* is also an ETS transcription factor, and the related gene set “GROSS_HYPOXIA_VIA_ELK3_UP” (*P* = 5.21 × 10^− 4^) might be relevant to LUAD survival. Two chemical compounds related gene sets were discovered in superset 1, “MARTINEZ_RESPONSE_ TO_TRABECTEDIN_DN” (*P* = 0.0015) and “CONCANNON_APOPTOSIS_BY_EPOXOMICIN_DN” (*P* = 0.0264). While both gene lists were derived from studies of other cancer types (e.g., HCT116 colon cancer cell-line), other studies have demonstrated the effectiveness of both Epoxomicin and Trabectedin in lung cancer treatment. Carfilzomib, which is a designed drug based on Epoxomicin, demonstrated anti-proliferative activity and resulted in prolonged survival in mice with SHP-77 small cell lung cancer xenografts [39]. There was only one study testing treatment with trabectedin on NSCLC patients, but no recommendation was given to use trabectedin as single agent treatment [40]. Thus, these two gene sets could be further examined to look for the biological relevance to LUAD.

**Table 6:** Top 15 gene sets in the highest ranked superset in LUAD survival analysis

Gene Set Terms	*P*-value^a^	*gsScore*	Weight^b^
CUI_TCF21_TARGETS_2_UP	1.30 × 10^− 4^	0.446	−0.211
RICKMAN_TUMOR_DIFFERENTIATED_WELL_VS_POORLY_DN	2.02 × 10^−4^	0.254	−0.201
GROSS_HYPOXIA_VIA_ELK3_UP	5.21 × 10^−4^	0.245	0.115
KAAB_HEART_ATRIUM_VS_VENTRICLE_DN	5.10 × 10^−4^	0.194	0.145
MARTINEZ_RESPONSE_TO_TRABECTEDIN_DN	0.0015	0.183	0.107
MITSIADES_RESPONSE_TO_APLIDIN_UP	0.2863	0.159	−0.171
KIM_WT1_TARGETS_DN	0.0064	0.146	0.081
ENK_UV_RESPONSE_EPIDERMIS_UP	1 × 10^−5^	0.143	0.077
SENESE_HDAC1_TARGETS_DN	0.8285	0.138	−0.162
SENGUPTA_NASOPHARYNGEAL_CARCINOMA_WITH_LMP1_UP	0.1411	0.129	−0.130
YANG_BCL3_TARGETS_UP	0.0299	0.126	0.163
GINESTIER_BREAST_CANCER_ZNF217_AMPLIFIED_DN	0.9507	0.124	0.132
CONCANNON_APOPTOSIS_BY_EPOXOMICIN_DN	0.0264	0.112	0.147
SPIELMAN_LYMPHOBLAST_EUROPEAN_VS_ASIAN_UP	0.0048	0.110	−0.051
RHEIN_ALL_GLUCOCORTICOID_THERAPY_DN	0.0154	0.107	0.051

^a^The *P*-value of gene set log-rank. ^b^The weight in the model corresponding to the connection of a gene set to the superset

**Table 7:** Top 15 gene sets in 4th ranked superset in LUAD survival analysis

Gene Set Terms	*P*-value^a^	*gsScore*	Weight^b^
SWEET_LUNG_CANCER_KRAS_DN	0.7304	0.780	−0.185
ZHANG_BREAST_CANCER_PROGENITORS_UP	0.0248	0.256	0.096
ROZANOV_MMP14_TARGETS_UP	0.1038	0.161	0.103
MONNIER_POSTRADIATION_TUMOR_ESCAPE_DN	0.0058	0.157	−0.117
ACEVEDO_FGFR1_TARGETS_IN_PROSTATE_CANCER_MODEL_DN	0.0988	0.154	0.114
YOSHIMURA_MAPK8_TARGETS_DN	0.0195	0.150	−0.126
DELYS_THYROID_CANCER_DN	0.0065	0.125	−0.079
SWEET_LUNG_CANCER_KRAS_UP	0.2762	0.122	0.141
OSWALD_HEMATOPOIETIC_STEM_CELL_IN_COLLAGEN_GEL_DN	0.0132	0.101	0.120
GROSS_HYPOXIA_VIA_ELK3_UP	5.21 × 10^−4^	0.100	0.058
WATTEL_AUTONOMOUS_THYROID_ADENOMA_UP	0.1555	0.096	−0.089
VERHAAK_GLIOBLASTOMA_MESENCHYMAL	0.7314	0.095	0.113
PHONG_TNF_RESPONSE_NOT_VIA_P38	0.0972	0.093	−0.121
RUTELLA_RESPONSE_TO_HGF_UP	0.7217	0.091	−0.055
IWANAGA_CARCINOGENESIS_BY_KRAS_PTEN_UP	0.0249	0.090	0.088

^a^The *P*-value of gene set log-rank. ^b^The weight in the model corresponding to the connection of a gene set to the superset

In 4th ranked superset, “IWANAGA_CARCINOGENESIS_BY_KRAS_PTEN_UP” (*P* = 0.0249) is a widely studied gene set to show the association with LUAD prognosis. The study that concluded this gene set observed the aberration in NSCLC with oncogenic form of *KRAS* and inactivated *PTEN*, in which condition resulted in shorter survival [41]. The gene set “ZHANG_BREAST_CANCER_PROGENITORS_UP” (*P* = 0.0248) shows the effect of progenitor cells in survival. Ooi et al. suggested that the presence of a putative tumor-initiating progenitor cell population in NSCLC is a biomarker with a worse prognosis [42]. *MAPK8* related gene set “YOSHIMURA_MAPK8_TARGETS_DN” (*P* = 0.0195) is also a potential prognostic associated gene set, while only one study implied indirect impact of poor prognosis due to MAPK8 repression [43].

From the two selected supersets, we already found some gene sets highly associated with LUAD survival, there are some novel prognostic gene set candidates that need to be further studied. In conclusion, superset results encompass survival-associated features and sort out the priority of potential prognostic gene sets.

### Improved survival reproducibility from supersets

To compare the reproducibility of survival results between the superset and gene set layers, we selected four TCGA data sets (BRCA, LUAD, SKCM, and LGG) to examine the reproducibility of GSAE. For each data set, we omitted genes that did not meet the criterion of *μ* > 1 and *σ* > 0.5. We next randomly split 60% of the data as the training set and the remaining 40% as the test set. After the autoencoder was trained on the training set, we obtained the superset outputs for the training and test sets. Median split and log-rank test were performed on training and test superset results to determine survival-related supersets and gene sets.

We assumed that the prognostic significant gene sets and supersets should be similar between training and test data. To evaluate the performance of gene set and superset results, we compared the significant gene sets and supersets obtained from training data and those from test data by Jaccard index. Furthermore, we used two population proportions z-test to examine whether supersets have greater overlap proportion in the training data, and the results are shown in Table 8.

**Table 8:** The statistical information of GSAE outputs between the training and test TCGA data sets of four cancer types

			Two proportion z-test		
TCGA data set	Superset Jaccard Index^a^	Gene set Jaccard Index^b^	Superset Proportion^c^	Gene set Proportion^d^	*P*-value^e^
BRCA	0.344	0.124	11 / 24	31 / 197	0.0002
LUAD	0.182	0.113	6 / 12	32 / 145	0.0150
SKCM	0.179	0.069	5 / 19	17 / 139	0.0485
LGG	0.483	0.475	29 / 45	299 / 481	0.3821

Supersets/gene sets with log-rank *P*-value < 0.05 were selected as prognostic significant sets. ^a^Jaccard index of significant supersets between training and test data. ^b^Jaccard index of significant gene sets between training and test data. ^c^Superset proportion: (# of overlapped significant supersets between training and test data) over (# of significant supersets in training data). ^d^Gene set proportion: (# of overlapped significant gene sets between training and test data) over (# of significant gene sets in training data). ^e^The *P*-value of z-test comparing superset and gene set proportions

In the largest data set BRCA, we found out that superset has much higher Jaccard index (34%) than gene set (12%), and the two overlap proportions differ significantly (*P* = 2 × 10^− 4^). In two other smaller data sets, LUAD and SKCM, superset (Jaccard Index ~18%) still outperforms gene set (11 and 7% for LUAD and SKCM, respectively; z-test *P*-value < 0.05). In LGG, because of the large number of prognostic significant nodes for superset and gene sets, both Jaccard coefficients are high (~48% for both superset and gene set) and the performance of gene set and superset is identical. To avoid the potential of sampling bias, we repeated the whole process in BRCA and LUAD several times, and we obtained similar stability measure (z-test P-value, data not shown). Overall, we concluded that superset has better reproducibility performance over gene set.

## Discussion

Same as other machine learning algorithms, the selective process of GSAE is an issue. Despite getting identical losses, different nodes (or gene sets) in different training may selective activated or de-activated (output value ~0) with the same training data. Take our study for example, we might obtain the same outcome (e.g. tumor subtype classification) of a dataset in the superset layer, but it is difficult to match superset between runs, and the top ranked gene set components in significant supersets might also be different, although highly relevant gene sets appear more frequent. This observation can be used to assess the significance of a given gene set or superset to a specific aim (e.g. survival association).

We also tried to understand the major cause of selective process in our model, and two possible factors were concluded – the dependency among gene sets in the CGP collection and the initialization of the model weights. Even though we tried to mitigate the dependency effect, many gene sets still share a subset of genes. In the model, we observed that some gene sets with zero values (deactivated) are highly overlapped with top ranked gene sets (activated). We assume that the information (member genes) of a zero-value gene set can be replaced by a combination of other gene sets. In addition, all weights in the GSAE model are randomly initialized. Due to the randomly initialized weights and dependency among gene sets, the model can reconstruct the input data through different gene sets, which results in the selective process of activated or deactivated gene sets. A better choice for independent or less overlapping gene sets could be Gene Ontology (GO) slims, a cut-down version of the whole GO. We might also alleviate this selective issue by assigning saved initial weights from a previous run or pre-trained weights of other data.

Another limitation of our model is the requirement of large sample size, which is a constraint for usual bulk RNA-seq experiments. However, the characteristic of single-cell RNA-seq (scRNA-seq) experiments, lower read depth with much larger sample size (near half-million scRNA-seq in some studies [44, 45]), perhaps is more suitable to be analyzed by deep learning methods. There are many advantages to examine scRNA-seq data using GSAE. First, scRNA-seq analysis with our model will not be restricted by statistical assumptions, where we can avoid dealing with the diverse statistical characteristics of single-cell data [46]. Second, we can directly determine the exclusive gene sets or GO functions of each identified subpopulation, without the need to find the representative genes of a subpopulation. With the support of other additional tools, analyzing scRNA-seq data with our model will be more thorough. For example, using only highly variable genes detected by scVEGs [47] will increase the diversity of subpopulations while lowering the variable dimension of the model. The HDBSCAN algorithm can cluster the multiple subpopulations of single-cell data precisely after t-SNE is applied. Overall, there is a huge potential using deep learning methods for scRNA-seq data analysis.

The concept of gene supersets not only provides better reproducibility, it also gives us a chance to understand the inter-dependency of gene sets. In this study we investigated the associations between significant supersets and gene sets. However, relations between those top ranked gene sets in the same superset has yet to be discussed. One possible solution is to find the corresponding input genes that have large contribution to a significant superset (by interpreting the weights in the first layer as the gene weights in each gene set), where we can further form a set of genes based on the superset. All these alternative approaches will guide our future study to bolster the biological functions of supersets.

## Conclusions

In this paper, we proposed a multi-layer autoencoder model with the incorporation of annotated gene set information. The model is capable of preserving crucial biological features of gene expression data in the dimension reduced superset layer. From the superset results, we have found out information such as tumor subtype differentiation and clinical prognostic significance. With the concept of superset, an unbiased combination of gene sets, we can improve the reproducibility of survival analysis, provide robust prediction of cancer subtypes, and indicate potential gene sets association of a disease. GSAE has the versatility to incorporate different gene set collection, discover different biological relevance, and analyze different kinds of gene expression data.

## Additional files

Additional file 1: **Table S1.** Top high impact gene sets of the four up-supersets and three down-supersets in the BRCA tumor subtype analysis. The highly overlapped collections of a gene set were determined by MSigDB (in the compute overlaps section). (XLSX 27 kb)

Additional file 2: **Figure S1.** The architectures of four neural network classifiers. (A) superset classifier, (B) gene set classifier, (C) 2-layer fully connected encoder network classifier, and (D) 4-layer fully connected encoder classifier. (PDF 328 kb)

Additional file 3: **Table S2.** Top 20 gene sets of the six prognostic significant supersets in LUAD survival analysis. (XLSX 15 kb)

## Abbreviations

BRCA: Breast invasive carcinoma
CGP: Chemical and genetic perturbations
CNN: Convolutional neural network
DAVID: The Database for Annotation, Visualization and Integrated Discovery
GO: Gene Onotology
GSAE: Gene Superset Autoencoder
GSEA: Gene Set Enrichment Analysis
HDBSCAN: Hierarchical Density-Based Spatial Clustering of Applications with Noise
IID index: Inter-Intra Distance index
LGG: Lower grade glioma
LUAD: Lung adenocarcinoma
MSigDB: Molecular Signatures Database
MWW: Mann-Whitney-Wilcoxon *U* test
NSCLC: Non-small-cell lung cancers
PanCan: Pan-Cancer
PCA: Principal Component Analysis
PDI: Protein–DNA Interactions
PPI: Protein–Protein Interactions
ReLU: Rectified linear unit
scRNA-seq: single-cell RNA-seq
SGD: Stochastic Gradient Descent
SKCM: Skin cutaneous melanoma
TCGA: The Cancer Genome Atlas
TPM: Transcripts Per Million
t-SNE: t-Distributed Stochastic Neighbor Embedding
