# Supplementary figures and images for: GSAE: an autoencoder with embedded gene-set nodes for genomics functional characterization
Source: BMC Syst Biol. 2018 Dec 21;12(Suppl 8):142. doi: 10.1186/s12918-018-0642-2 (PMC6302374; doi:10.1186/s12918-018-0642-2)

A

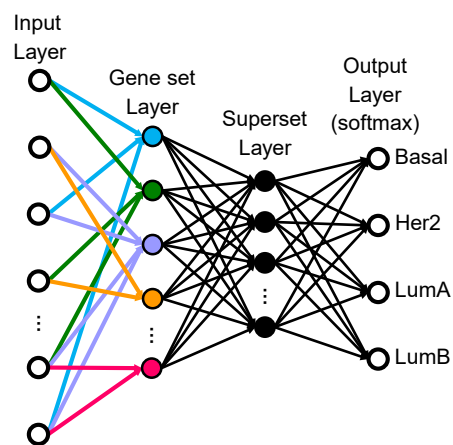

B

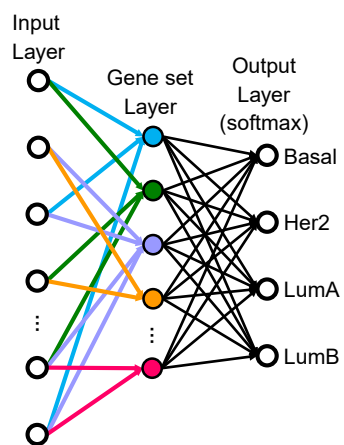

C

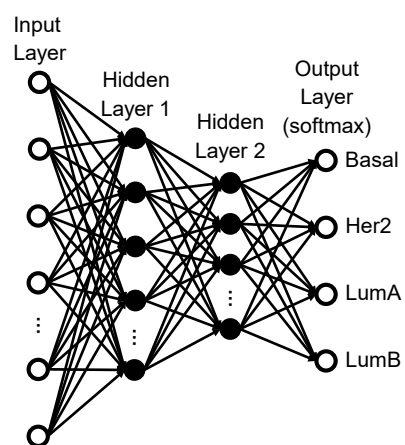

D

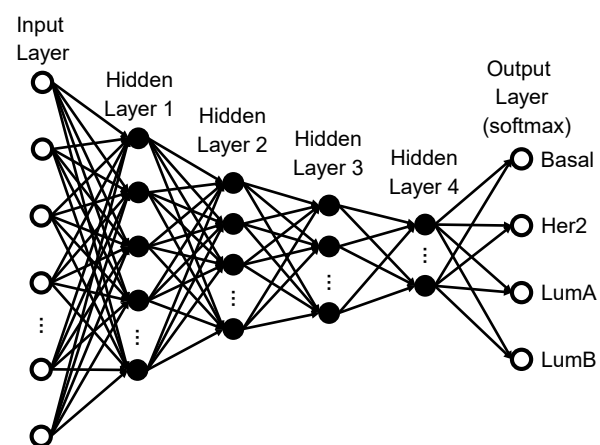

Supplement: Supplementary file 2 — Figure S1. The architectures of four neural network classifiers. (A) superset classifier, (B) gene set classifier, (C) 2-layer fully connected encoder network classifier, and (D) 4-layer fully connected encoder classifier. (PDF 328 kb) [file 12918_2018_642_MOESM2_ESM.pdf]
